# Communication and building social capital in community supported agriculture

**DOI:** 10.5304/jafscd.2022.121.009

**Published:** 2022

**Authors:** Ella Furness, Angelina Sanderson Bellamy, Adrian Clear, Samantha Mitchell Finnigan, J. Elliot Meador, Susanna Mills, Alice E. Milne, Ryan T. Sharp

**Affiliations:** a Sustainable Places Research Institute, Cardiff University; 33 Park Place; Cardiff, CF10 3BA, UK; b Department of Applied Sciences, University of the West of England at Bristol; Frenchay Campus, 6 Coldharbour Lane; Bristol, BS16 1QY, UK; c NorSC Lab, Faculty of Environment and Engineering, Northumbria University. Adrian Clear is now at School of Computer Science, National University of Ireland Galway; d NorSC Lab, Faculty of Environment and Engineering, Northumbria University; Newcastle upon Tyne, UK; e Rural Policy Centre, Scotland’s Rural College; Edinburgh, UK; f Population Health Sciences Institute, Newcastle University; Newcastle upon Tyne, UK; g Sustainable Agriculture Sciences Department, Rothamsted Research; Harpenden, Hertfordshire, UK; h Sustainable Agriculture Sciences Department, Rothamsted Research; Harpenden, Hertfordshire, UK

**Keywords:** Alternative Food Networks, Civic Agriculture

## Abstract

Community supported agriculture (CSA) schemes (programs) provide an alternative means for obtaining produce, through direct purchase from farms. They are also often driven by a vision of transforming the current mainstream food system and seek to build a community of people who support this vision. Social capital refers to the networks and ties between people and groups and the impact of these ties on access to influence, information, opportunity, and ability to organize. Social capital is built by CSAs and helps foster and stabilize the grassroots agricultural innovations that are needed for the development of sustainable food systems. Using the concept of social capital, we studied communication methods of four CSAs in the UK, examining the interactions between CSAs and their members and within each of their membership groups. We carried out in-depth interviews with 49 CSA members to establish what interactions they had with their CSA and with other members, and analyzed our data thematically to identify the characteristics of interactions that were important to participants. We consider how our research may benefit CSA organizations by enabling them to learn what their members want and to learn about the varied ways in which members conceptualize their experiences of community derived from their membership. We found that the various CSA communication strategies, which consist of frequent and varying virtual and face-to-face interactions, are able to promote development of both bridging and bonding social capital. Overall, there is a desire for social connection in CSA memberships. Furthermore, in CSAs where members can interact easily, there is potential for CSA membership to provide members with communication that is important as a source of both knowledge and social connection. CSAs can maximize both social capital and member satisfaction by using a range of communication media and methods to meet their members’ circumstances and preferences.

## Introduction

The urgent necessity of transforming food systems for reasons of sustainability, food security, and health has been well documented (IPES–Food & Nourish Scotland, 2021; Willett et al., 2019). As with most environmental issues, government, industry, and technology all have a role to play, and a range of potential avenues exist for generating change in food systems (Pralle, 2006). One potential means of sustainable food system transformation, which forms the focus of this study, is community-based innovation, which often aims to relocalize food systems by shortening supply chains, building social capital, and creating sustainable income sources for small-scale farmers (Gleissman et al., 2018). In this study, we focused on the role of the effective building of social capital, by investigating the specific communication strategies that enable community supported agriculture projects (CSAs) to develop social capital.

## Community Supported Agriculture

A CSA is a partnership between farmers and consumers in which the responsibilities and the risks and rewards of farming are shared (Community Supported Agriculture Network UK, 2022; European CSA Research Group, 2016). A wide variety of governance arrangements exist, but usually the consumer offers something more to the CSA than just a straightforward exchange of money for produce. For example, the consumer may contribute labor, take some financial risk by investing in the CSA, play a part in decision-making, and/or accept a variable share of produce proportionate to the success of harvests. Accordingly, participants in CSAs are often referred to as members rather than customers.

The first CSA in the UK was established in 1994, and in 2020 there were 179 CSAs, although many are in the early stages of setting up. The CSA Network UK was launched at the end of 2013, and currently represents 111 of these organizations (Suzy Russell, Community Supported Agriculture Network UK, personal communication, September 17, 2020). As is common in many small and precarious sectors, it is difficult to estimate accurately how many CSAs are operating at any time, and thus how many people are members (European CSA Research Group, 2016). The number of members per CSA in the UK ranges from less than ten to hundreds, with an average of 87 members (European CSA Research Group, 2016).

### CSAs and Food System Transformation

Though CSAs currently represent a small proportion of agriculture in the UK, their potential as agents of change in enabling a more sustainable food system is significant. CSAs can be viewed as part of a wider set of community infrastructure projects which includes consumer co-ops, solidarity buying groups of local and organic food, and collective urban gardening initiatives forming Civic Food Networks (CFNs) (Renting et al., 2012) or civic agriculture (Kaika & Racelis, 2021). These innovations are a response to lack of communica-tion between food producers and the general public in the UK, which has long been recognized as a problem that entrenches public alienation from the way their food is grown and processed (Duffy et al., 2005; Opitz et al., 2019). In selling direct from farm to consumer, CSAs seek to strengthen the interactions between consumers and their local food supply (Opitz et al., 2019). Emerging evidence strongly suggests that CSAs can positively impact members’ understandings of food systems and influence their food behaviors and health outcomes (Allen et al., 2017; Rossi et al., 2017). Members often gain knowledge of seasonality, cooking, nutrition, cultivation practices, and farmers’ perspectives (Opitz et al., 2017). CSAs can also affect environmental change by fostering sustainable behaviors, providing food with low environmental impact, and building social capital and resilience at the regional level (Saltmarsh et al., 2011). Fostering social capital is one method of increasing the socio-political capabilities of alternative food networks and their ability to transform existing entrenched unsustainable food systems (Mert-Cakal & Miele, 2020). The unique CSA membership structure has potential for developing social capital by reconnecting consumers and producers, and it is this aspect of CSAs upon which we focus.

### Social Capital

The concept of social capital has multiple origins, with the writings of Bourdieu (1984), Coleman (1988), and Putnam (2001) central to its development. Putnam defines social capital as the “social norms and networks that enhance people’s ability to collaborate on common endeavors” (2001, p. 135). Social capital is sometimes metaphorically described as the glue that holds groups together or the grease that enables people to get things done (Kay, 2006). Building on understanding social capital as communication and linkage between people, social network theory examines the types and amounts of relationships (or “ties”) that people and groups have with each other, and the impact of these ties on “influence and information, mobility opportunity, and community organization” (Granovetter, 1973, p. 1360). Three kinds of social capital are generally agreed upon in the literature. Bonding social capital is characterized by intimacy and the development of strong ties, often around shared characteristics. It enables reciprocal support, but it can also limit the expansion of trusting relationships beyond a niche community. Bridging social capital is usually characterized by weaker ties and is created when two otherwise unconnected individuals are linked. Bonding and bridging capital increase the capacity for change and adaptation within communities. A third kind of social capital, “linking” capital, involves the development of connections between groups or individuals of different social status. Linking social capital can be thought of as connections between people with different levels of power within society. These connections can create opportunities for change by creating dialogue between innovators and groups and individuals with influence and resources. Groups which create social capital also create entrepreneurship and innovation and encourage initiative, responsibility and adaptability, which are all required to meet the challenge of bottom-up transformations of the food system (Glowacki-Dudka et al., 2013).

### CSAs and Social Capital

Identifying effective and efficient methods that enable CSAs to build social capital is key to supporting the creation of social movements to promote sustainable food. Research suggests that increased opportunity for communication and participation enhances the commitment of CSA members to the ideals of alternative food networks and to the CSAs themselves (Haney et al., 2015; Opitz et al., 2019). Increased communication enables CSA members to develop trust in the community formed by the farm staff and members (relational trust) and in the organization itself (institutional trust). Previous research suggests that relational trust is dependent upon face-to-face contact (farm visits, collecting produce directly from the farm) as well as digital communication such as social media, email, and use of online organizational tools such as Doodle polls (Aissaoui et al., 2017; De Bernardi et al., 2020). Hands-on food-growing work with the CSA may also play an important role in ena-bling members to use all their senses, deepen their understanding of the reality of agricultural work, and build their understanding of the organization (Aissaoui et al., 2017; Carolan, 2007).

While essential to the mission of transforming the food system, research has found that building social capital nevertheless can be a drain on CSAs’ limited resources (Galt et al., 2019; Mert-Cakal & Miele, 2020). As observed by Rossi et al. (2017), researchers need to establish what kind of member engagement is required to create a thriving and innovative food system. Our research aims to fill this gap in understanding, to determine where time invested in communication and outreach to CSA members may derive the greatest social capital dividends. We examine how four CSAs in the UK communicate with their members, how their members interact with each other, and what value members place on this communication. Crucially, we look at what interaction members want and why, to enable CSAs to focus their efforts for maximum effect. By examining the kinds of participation that CSA members engage in and value, our research aims to provide knowledge that can enable CSAs to scale up and play a more significant role in enabling food system transformation. It can also contribute to the development of the CSA sector by creating a data base for both CSAs and policymakers who are seeking to support developing alternative food networks and transforming entrenched unsustainable food systems.

## Methods

This article presents four CSA case studies. Data were collected via in-depth interviews with CSA members to build communication profiles of each of these CSAs. We asked three research questions:

1. How do CSAs interact with their members?
2. How do CSA members interact with each other?
3. What interaction do CSA members want and why?

We recruited 49 CSA members who had joined a CSA program in the 12-month period prior to interviewing in the summer of 2019. We selected relatively new members, rather than those who have already built social capital, to understand how participants responded to different opportunities to build social capital. The participants were members of four CSA organizations operating in Wales and England. We chose four case study farms that represented different CSA business models, to capture as much variability in CSA operations as possible while still enabling in-depth study of each case (Table 1).

CSA 1 was a family farm in a rural area in South Wales; they were diversifying, had a vision for building a local food culture, and as part of this goal they began a vegetable box program. CSA 2, in a rural area, had an established vegetable box program run as a workers’ co-operative since 2018, with a vision of supplying organic vegetables to local residents. Most member households were from a nearby city. CSA 3 was a not-for-profit social enterprise focused on low-carbon production methods and whose member households usually contribute both labor and money to pay for their share of the vegetable harvest. CSA 4 was a cooperative run by its members that developed from public conversations about changing unsustainable food systems as part of the Transition Towns movement (https://transitionnetwork.org). CSAs 1, 3, and 4 shared the objective of building a community around their stated vision.

We incentivized participants to join the study by offering one free vegetable bag from the host CSA or a financial equivalent, depending on the preference of the CSA hosts. Initial contacts with members were made either face-to-face at the CSA sites or by email via the host CSA. Interviews were carried out face-to-face or by phone. We emphasized data about communication between CSA and members: frequency of contact, media used, topics discussed, and intra-CSA communication. We also examined CSA member expectations regarding interactions with the CSA and its members. The interviews contained some questions that give an overview of the mediums of communication used, the frequency of communication, and the topics discussed, and other questions more in-depth and which were analyzed using the thematic approach described below. It is important to note that our sample sizes per CSA are not sufficient to warrant the use of inferential statistics. CSA 1 had 21 members at the time of data collection and 15 participated in our research, CSA 2 had 66 members and 6 participated, CSA 3 had 65 and 11 participated, and CSA 4 had 120 members and 14 participated. In addition to the interview data represented here, we also discussed the research with representatives of the CSAs and examined other sources of information to enable a degree of triangulation of the interview data with other sources. For example, we corroborated data gained through interviews by subscribing to CSA newsletters (or requesting copies from CSA representatives) and observing CSAs’ publicly available social media activity. The study methods were approved by the Cardiff University School of Geography and Planning Ethics Committee.

After interviews were recorded, transcribed, and anonymized, we applied a coding procedure, derived from Strauss (1987), Miles and Huberman (1994) and Coffey and Atkinson (1996), that involved filing all the data (using the software package NVivo) and identifying themes. Initially we revisited the three research questions and coded any data relevant to them. For example, any data that mentioned communicating in a particular medium was coded as that medium: i.e., comments about WhatsApp were initially coded as “WhatsApp.” The second stage of coding involved identifying “in-vivo” themes present in the data and coding them accordingly. These were strong themes that emerged from the data but were not necessarily apparent before the study began, either in our research questions or previous literature. The thematic analysis was carried out iteratively until no new themes arose, data saturation was reached (Fusch & Ness, 2015), and the definitive findings emerged. Below we present an overview of communication within CSAs, and then explore in more depth the themes that arose, illustrating our findings with extracts from transcripts.

## Results and Discussion

### Communication Between CSAs and Their Members

**Figure 1:**
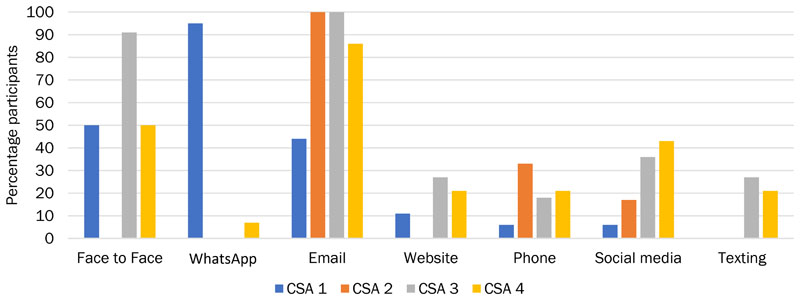
Percentage of Participants in Each CSA that Used the Communication Media Listed

**Figure 2:**
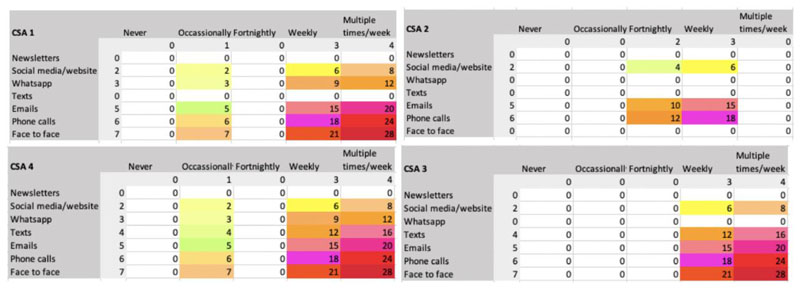
Bridging and Bonding Social Capital Across the Four Case Studies Lighter colors indicate bridging capital and darker colors indicate bonding capital.

We found that each CSA had a different communication style (Figure 1). CSAs also used different mediums of communication, with varied amount of contact with members and topics of communication. As a result, the degree to which members were able to become familiar with the farmers, growers, or staff of the CSA varied. CSAs 1, 3, and 4 developed relationships with their members, whereas CSA 2 staff were more inaccessible. CSA 2 had the least amount of communication via the smallest number of mediums, concentrating on email. There was less contact between CSA 2 members and their CSA than in the other CSAs, usually via emails about the produce. While participants expressed satisfaction with this level of communication, there was far less social capital (bridging or bonding) built in CSA 2 (Figure 2). However, since the vision of the CSA is limited to pro-viding organic vegetables to local households, the limited amount of social capital is not likely to be viewed as problematic by the CSA itself. This is especially so, considering that CSA 2 is operating at capacity and often with a wait list for people wanting to join.

Figure 2 illustrates how bridging and bonding social capital are fostered for each case study. We considered the nature of the activity (one- or two-way communication flows, virtual or face-to-face communication) and the frequency of the activity to determine how it contributed to building bridging and bonding social capital. Moving down the rows and across columns in Figure 2, activities are likely to move from building bridging to bonding capital. Figure 2 shows that while CSA 1, 3, and 4 differ in their communication strategies, each is building both types of capital in multiple ways.

CSA 1 and 2 had a relationship with their members generally resembling a typical transactional relationship: they predominantly communicated about the produce itself, how to use it, and arrangements for obtaining it. There was a clear line between the organization and its customers. However, whereas CSA 2 had a solely transactional relationship with members, CSA 1 saw building relationships with customers to be an objective. At CSA 1 there were some members who became friends of the farmers, moving beyond bridging social capital to bonding social capital. Face-to-face communication with the farmers was a key reason for many members’ enjoyment of the program and served as an important source of bonding capital.

**Figure 3:**
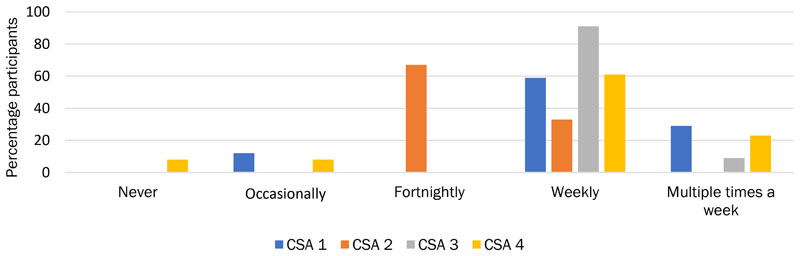
Frequency of CSA Contact with Members Across the Four CSAs

CSAs 3 and 4 were usually in touch with members once a week or more (Figure 3). Their relationships with members were more collegial, with discussion about the produce itself augmented with discussion about the logistics and tasks involved in growing produce, the problems involved in running the CSA organization itself, and development plans.

CSAs 1, 3, and 4 provided opportunities for communication between members. Communication was influenced by the governance arrangements of the CSA and arrangements for accessing the farm and collecting produce. For example, CSA 1 had no arrangements for members to be involved in decision-making, whereas 3 and 4 had inclusive governance models. This largely explains why members of CSA 1 talk primarily about vegetables and recipes whereas CSAs 3 and 4 also discussed practical and administrative problems in managing the CSA as well as agriculture and the environment more generally. The CSA collection arrangements, accessibility of the farm or growing site, and volunteering opportunities dictated how much members interacted and built bonding social capital through shared interests.

**Figure 4:**
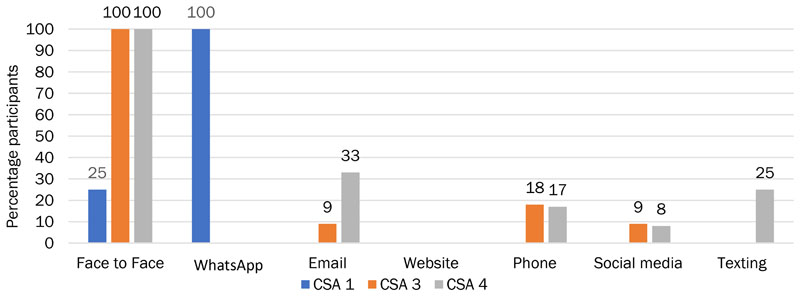
Type of Media Used by Participants to Communicate with Other Members

**Figure 5:**
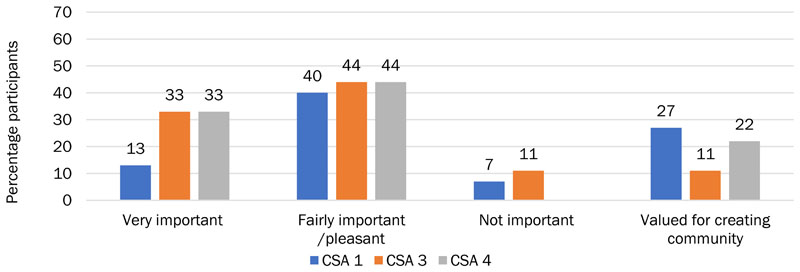
CSA Members’ Evaluations of the Importance of Their Interactions with Other Members

CSA 1 members were predominantly using WhatsApp to communicate with each other, with a quarter of the members also communicating face-to-face (Figure 4). Just under a third of participants had pre-existing friendships with other members of the CSA (28%) and thus had bonding capital that existed prior to CSA membership and outside the CSA. At the other end of the spectrum, a group of “arm’s length” members communicated once or twice with other members or not at all. There was a group of members (13%) who were more intimate and socialized together and other smaller groups who were more involved in the CSA and discussed volunteering arrangements and practical tasks. Members of CSA 1 were more likely than other CSAs to value the communication they engaged in for creating a sense of community (40%); however, they treated it lightly, finding it pleasant or fairly important rather than very important, indicating that CSA 1 might have stronger bridging capital than bonding capital (Figure 5).

All CSA 3 members who communicated with each other reported doing so face-to-face; 18% also spoke on the phone, and 9% via email and social media. There were fewer independent friendships within the group than at CSA 1 or CSA 4, although 18% of CSA 3 members had pre-existing friendships with other CSA members. Members often socialized, discussing practical problems, agriculture and the environment, events and plans, and the produce itself (Figure 3). A third of CSA 3 members thought their communication was very important, the remainder thought the communication was pleasant and fairly important, and smaller proportions valued it for building community or did not view it as important (Figure 5).

**Figure 6:**
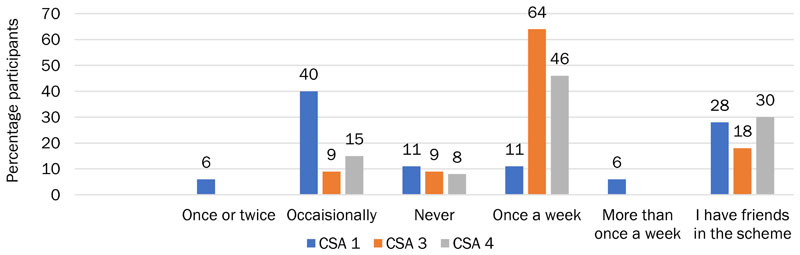
Frequency That CSA Members Were in Contact with Each Other

We found that CSA 4 members predominantly communicated weekly (46%, Figure 6). In addition, almost a third (31%) of participants had pre-existing friendships with other members of the CSA. Of those CSA 4 members who communicated with each other, 83% engaged in general socializing with other members, with half of communication reported as socializing. Other than social conversation, 33% of members discussed the CSA and wider topics to do with agriculture and the environment, and 17% discussed practical tasks. Similar to CSA 3, a third of CSA 4 members thought their communication was very important; the remainder thought the communication was pleasant and fairly important and valued it for building community (Figure 5). This indicates that CSA 3 and 4 have built stronger bonding capital than bridging capital, as reflected in Figure 2.

## What Interaction Do CSA Members Want and Why?

### Face-to-Face Interactions

As members reflected on the communication they had with the CSA and with each other, it became clear that participants had a variety of needs and preferences about communication. Across all CSAs, the value of face-to-face communication was a strong theme. Although there was some indication that WhatsApp provided opportunities for creation of bonding social capital (Figure 2), more prospects seemed to be available face-to-face. Face-to-face communication varied in intensity: from almost incidental, such as a casual “Hello, how are you?” as participants weigh out their vegetable share at a collection point, to longer bonding social capital–building opportunities such as working alongside other volunteers in the fields every week. Communication that occurs during temporary close proximity, such as serendipitous meetings with fellow members at collection points, emerged as a key theme in the data. Members frequently talked about these meetings and the value they added to their everyday life.

The value of social connection gained through volunteering was a strong theme in the data, irrespective of the CSA that hosted the participant. Volunteering tended to be something that CSA members often did weekly, for two hours or more. This ongoing collaboration provided the opportunity for communication about the practical aspects of the job as well as for broader social communication to take place:

I suppose there’s two levels of information. One is like function, you know, if you’re working on tasks together. … And the other is, you know, the general chitchat of social communication really, like opinions about the world. (CSA 3, Participant 3)

Reasons given by CSA members for volunteering include valuing the opportunity to be outdoors in nature, working with their hands, feeling the achievement gained from manual work, and having free time to support the vision of the CSA. These volunteers were focused on the work, and social capital developed as an unintended consequence of volunteering. Volunteering was an excellent way of developing social capital among people who were less outgoing: there was less pressure on them to perform socially, as the social interaction was a side effect of the work:

It feels easier every time, I’m an introvert … and so it’s really nice. James [the grower] particularly is so friendly, and very easy to chat to. Yeah, and again particularly on harvest days when I’ve been paired with someone to do a task. That’s been really nice as well, because it’s a way to get to know people. (CSA 3, Participant 11)

When you’re … doing something, it’s a nice relaxed way … of communicating … isn’t it? Because it’s OK when you’re busy … it’s a way to get to know people. (CSA 3, Participant 3)

These quotes clearly demonstrate the mental health benefits of volunteering, a strong theme emerging from the data. The available social connection seemed to be a solace, providing a safe situation for CSA members to challenge themselves. The work provided a buffer between these volunteers and the pressure of social interaction. It thus became a vehicle for building bonding capital even for those who experience stress during social interactions:

I have quite bad social anxiety. … I am not the sort of person that would easily arrange to meet someone for coffee or just phone them up for a chat … Just talking to people, freestyling it, I’m not very good at that. … It really triggers my anxiety, which is one of the reasons I really like the farm is because I feel quite safe. Because there’s jobs to do, if I start feeling anxious … worrying about having something to talk about or worrying that I’m talking too much, I can just redirect myself back into the job that I’m doing. And I can then sort of regroup. So yeah, I haven’t ventured into socializing or chatting to anyone outside of the actual visits. … It’s something I will work on. That’s my … long-term project … to sort of build my confidence up again. (CSA 3, Participant 8)

For other CSA members, the opportunity to socialize was a significant part of why they volunteered. Wanting to grow food in a community, rather than managing an allotment or garden by oneself, was a central motivator for many volunteers, as with a participant who looked forward to meeting different people at the growing site:

[There have been] different phases … in winter it was quite quiet and similar people, a handful of people. … Now it’s summer and as the project has grown a bit, there are more different people coming … coming here … you’ll just wonder “who will be turning up today?” so it’s an additional motivator. (CSA 4, Participant 1)

While volunteering represents a time-intensive form of interaction to build bonding capital, picking up vegetables at collection points provided weekly face-to-face opportunities for building relationships based on short but valued conversations:

If I go to pick up the veg it is probably about ten minutes to a quarter of an hour. I do tend to stop and talk to either Jill or Brian. They’ve now sort of become friends more than somebody just that I buy veg off. I’ve got involved with them and their families. And so you get to know them really well. And they’re a lovely couple. (CSA 1, Participant 6)

CSA managers purposefully created this opportunity to build social capital and create social value through their programs. For example, one of our CSAs aims “to encourage community engagement in the growing, consuming, education and celebration of local, ecological and seasonal produce,” to “share knowledge and expertise to educate and enable others to benefit from our experi-ences” and to “co-create a viable community with a focus on social dividend, contribution and sharing” (CSA 3). Facilitating communication between members is part of this, and the casual conversations when collecting produce is something their membership enjoys:

Last year, Brian would leave the bags out and then we would just go and … quite often we wouldn’t see anybody. Whereas now this year … there has always been Brian or Jill. … And so I get to know Jill. … It’s nice to have those chats as well. So I actually look forward to going and collecting my veg on a Thursday. (CSA 1, Participant 3)

Members often regard face-to-face meetings as a way of building community as well:

[Collecting the veg] is something I like. And it’s funny because actually our neighbor … she’s 65 now, she’s retired and so we are sharing who goes to get the veggies. She goes one week, then we go. … [It affects] the sense of community inside our neighborhood. So yeah … I really enjoy going there. It’s a totally different experience from going to the super-market. … You chat to people. (CSA 4, Participant 4)

Volunteering tends to build bonding capital, while interactions occurring during vegetable bag pick-ups can help to support bridging capital. While both types of activities provide face-to-face contact, they build different kinds of social capital within the CSAs.

Falling between these two kinds of face-to-face interactions are group events, which tend to be less regular and more varied. Coffee mornings, farm tours, lambing, tractor rides, music events, and seasonal celebrations were mentioned as ways through which participants met others. A participant illustrates how events can build relationships and result in either bridging or bonding social capital:

I went along to that coffee morning and met some really nice people. You know, you start a conversation and there are similar interests and, you know, things organically grow with time. The relationships. I am not desperately trying to find new connections with people. That will happen, life makes that happen. (CSA 1, Participant 10)

### Virtual Communication

Although at the time the data were collected WhatsApp was only significantly used by one CSA, it was clear from this case that it was a useful tool for creating bridging capital, by communicating novel information between people who otherwise would not have spoken. On WhatsApp ties were weak to the extent that people may not know who they are communicating with:

They … take photographs … of what they’ve done and … put a link to the recipe … that’s quite good actually. I quite like that. I don’t know who she is … but she calls herself Organic Iris and she puts lots of recipes and things. I see the post, but I don’t know who she is. (CSA 1, Participant 1)

Members could participate as little or as much as they liked in the WhatsApp group, tailoring their interaction to the level they were comfortable with, as described by one participant:

WhatsApp is pretty good, because it’s a group conversation. You can dip in when it suits you. But then you get to see what everyone else had said as well. It’s very inclusive actually. That’s where technology is wonderful. So you can have a group conversation when it suits you. We all have busy lives, I think that’s what’s helpful about it. (CSA 1, Participant 13)

Below are extracts from interviews in which participants described meeting their different needs via the WhatsApp group. One participant used it a resource for recipes, emphasizing that the volume of communication needs to feel manageable.

With WhatsApp, you can be involved in so many groups, you don’t want to, you know, say you have overload and … you go back to your phone with 100 messages, that would be a bit much, I think. I think it’s a nice level, really, it’s not too much. There’s just a little bit of recipes or, you know, some information. (CSA 1, Participant 4)

Another participant describes the beginning of the development of bonding social capital, as participants get to know each other and share more of their lives:

It can be as simple as “Oh we don’t need the bag this week,” different orders. Or it can be “What’s in the bag this week?” so I can prepare a bit ahead. Often, it’s “What is this vegetable and what do I do with it?” so recipes are shared on WhatsApp. That’s it really: practicalities, recipes, advice … and we have a laugh as well … Someone sends a message like “It’s been one of these days, I’m having a glass of wine”—usually the farmers when they’ve been out in the rain … and photographs, he sends some lovely photographs. Herding sheep, ploughing fields. Yeah, that’s nice. … (CSA 1, Participant 13)

There was some indication that WhatsApp conversation did not work for all participants, however. Some people just wanted their vegetables without any added social capital:

I … and someone else who was on the [WhatsApp] group, do find it irritating when some people are “Uh, look what do I do with this pumpkin?” (mockingly) and you go like “Go and Google for goodness’ sake.” (laughs) … You know, I don’t need people asking for recipes and stuff. Use your brain, please. To me it was just veg grown on a farm. You pay for them: thank you very much. Goodbye. (CSA 1, Participant 19)

Overall, the ties created by the largely WhatsApp-based communication between members of CSA 1 created a valued sense of community among members (Figure 5).

Other forms of social media, such as Facebook and Instagram, were also valued and effective (Twitter was only mentioned by one participant). For some participants, social media was the main way that they communicated with the CSA and with other members; they also saw it as a form of outreach or as a way of supporting the farm:

The main interaction I have is on social media, because they’re quite active on Facebook now. So I want to sponsor their posts, and I also try and get my friends and family to sign up. So I, you know, post pictures of what I’ve got from the farm that day. And the meals that I’ve made with my share and put that out on Facebook. (CSA 4, Participant 8)

When I cook their food, I try and share it on Instagram, if it looks particularly beautiful, ‘cause I can think of a lot more people to shop at the farm. I wanted to say to Jill and Brian, “Look how grateful we are with what you are doing.” You know, we really love your produce. (CSA 1 Participant 15)

In addition to social media, email newsletters were valued by the members. They were often the central communication tool. CSA 2 had a newsletter only a few sentences long, whereas CSAs 3 and 4 had newsletters administered via online email marketing software with content about upcoming events, the welfare of the organization, the produce, recipes, other relevant local events or programs in which the CSA was participating, and calls for volunteers. A newsletter might seem to be a less sophisticated way of communicating than social media, but it was reliable and consistently read by participants, who gained insight and information about their CSA, even when they were only receiving a few sentences every week or fortnight:

I think it’s made me more aware of how, like, a wet summer or a dry winter, like, the impact that that can have on a particular veg. And they put that in the newsletter, you know, if, if something hasn’t come through for them, they’ll kind of say, you know, due to this particular spell of weather or whatever. Yeah, it’s made me more aware. (CSA 2, Participant 13)

The face-to-face and virtual interactions met participants’ desire to be part of a community, or to contribute toward a community, which was the strongest theme that emerged from the data. Members understood community building to be anything from the act of buying from a local farm rather than a supermarket to being a volunteer or socially active member, both face-to-face and online. *Not* wanting more social interaction via the CSA was also a recurring theme, and it is important to recognize that some members were not interested in developing community or social capital as part of their CSA membership. Some described themselves as “not being terribly social” (CSA 3, Participant 4); others were already involved in other communities:

We do have very packed social calendars for the kids, with the community around the school. … The principal reason that we’re involved [in the CSA is] the fact that I don’t want to be doing any harm with our veg buying. … That’s enough for me. (CSA 2, Participant 2)

Some had enough friends and not enough time to participate in additional CSA-connected relationships. Still others were using their involvement to spend time with particular people to deepen or improve existing important relationships:

The interaction I do like is with my daughter. [Volunteering is a] really nice, wholesome … active and involved and enthusiastic, and we’re learning together. … That interaction is probably my priority. And if I was socializing too much, you end up chatting to the other person and not relating to my daughter, which isn’t what I want. (CSA 3, Participant 5)

Each CSA had different kinds and amounts of social capital stemming from their different organizations. CSA 2 communicated almost solely through broadcast emails and had very little social capital associated with their membership; their members only rarely communicated with each other or with the farm, but this was driven by their primary objective, to provide organic vegetables, and therefore social capital was not required to achieve their objective. CSA 1 had more opportunities to communicate and develop bridging capital between people who may not have communicated otherwise. The membership group did not have enough communication to develop its own identity or bonding capital; the bonding capital that existed was exclusively between the pre-existing friend groups. CSAs 3 and 4 had more members who regarded communication with the CSA and other members as very important; both CSAs had a full suite of opportunities for their members to communicate, and which were used most frequently, from established volunteering schemes to social media. The most bridging and bonding social capital was being built in these CSAs.

Members of CSA 3 and 4 reflected on the bridging social capital that developed through their experiences at the CSA, first through intergenerational communication while volunteering, and second through meeting people from different backgrounds:

You have … a nice mixed age range of people, people with families, people with children. We both have a child of our own, but Bill’s daughter is in France, my son is in Manchester, but we don’t see them as much as we’d like, we don’t have any grandchildren. And it’s quite nice to see people in different ages and, you know, friends who are not the same age. (CSA 3, Participant 7)

You’d meet a different group of people certainly to what I normally meet, which is interesting, to some extent eye-opening. So yeah, you meet a different group of people to what you normally, to what I normally meet. I mean, there’s not many other doctors here. (CSA 4, Participant 1)

In contrast, a participant gave an example of bonding social capital, in which connections between people who are “like-minded” develop:

The people who are part of [CSA 4] are fairly important, not massively. I hope that one day it will be, I hope that I carry on being friends and sort of make deeper friendships, because … so many of them are people who, you know, they’re really like-minded, and I like my conversations with them, I get a lot from it. So, you know, in the future, I would hope that some of those would become good friends. Rather than sort of, you know, liked acquaintances. (CSA 4, Participant 8)

A participant reflects on the building of bonding social capital and the value of making connection with people who are different from oneself:

[CSA Membership is a] very nice way of getting to know people. You feel the same sort of wavelength. Though I realize one should also try not to just remain in one’s bubble … I’m constantly being reminded. (CSA 4, Participant 15)

This bubble that Participant 15 mentions is something important to acknowledge, as we found that our CSA members were overall more affluent and of higher socio-economic status than the UK general population. Lack of diverse socioeconomic representation means that there is a low possibility for creating linking capital. Our analysis found no examples of development of linking social capital. A member reflects on what she described as the “middle class” nature of CSA membership:

One of the things I would say, I think it’s quite a middle-class thing. So a lot of, it’s not cheap I don’t think. And … lots of people who are there, they’re often in health professions, or education. And so, you know, when you’re next to them, you’ve got quite a bit in common to chat about. So I have done that a few times. But you are, well I think you are with fairly like-minded people, or that is who I’ve bumped into. (CSA 3, Participant 3)

While they did not form part of the case studies, there are a growing number of CSAs that are experimenting with implementing solidarity models (Verfuerth & Sanderson Bellamy, 2022), as a reaction to the increased number of households experiencing food insecurity during the COVID-19 pandemic. These activities could serve to increase the opportunity for building linking social capital. As these alternative food networks include transforming the current dominant food system among their objectives, input and participation from people across socioeconomic levels will be required.

## Conclusion

By highlighting how different communication strategies build social capital, the research presented here can support further efforts within the CSA sector to generate transformative food system change. Our study indicates that there is variation in the way CSAs build social capital, leading to differences in the types that are built. In CSAs where members can interact easily, there are social and informational benefits, developed through both bonding and bridging capital. Preference and circumstance play a large role in the connectedness of individual CSA members, as does the particular CSA vision for change. Bonding social capital emerges from frequent face-to-face interactions, requiring an investment of time from participants and from CSA managers to organize. Some CSA members were happy with the relatively weak ties generated through WhatsApp communication, but most valued the opportunities to connect face-to-face at collection points and through volunteering.

To maximize social capital, CSAs should use a range of communication media. Social media, WhatsApp groups, face-to-face collection points, and volunteering opportunities can meet a range of their members’ circumstances and preferences. CSAs seeking to maximize social capital efficiently can do so with just a few sentences in a regular email. Setting up a WhatsApp group would likely be well received and enable easy communication between members. Further, we suggest asking members to volunteer to write the newsletter, since many participants were eager to do so. Efficiently building social capital, however, does not translate into achieving food system change. When deciding their communication strategies, CSAs need to consider their objectives and vision for change. Different types of social capital are required to achieve transformation; while bonding capital is important for creating an engaged and committed community, bridging capital enables greater reach beyond the immediate community, enabling change to ripple through fringe communities and thereby creating the conditions for transformation. Further research should explore how different types of social capital created by CSAs can translate into wider food system transformation, as suggested by Mert-Cakal and Miele (2020). Additional research is also required to understand if diversification of CSA membership can promote linking capital and the possibility of building a more representative food movement. Overall, in our study, we found that there was a hunger for social connection within CSA memberships, with desire for developing community a theme that was dominant throughout our data. We conclude that CSAs are fertile ground for building social capital to generate food system transitions.

## Figures and Tables

**Table 1:** Characteristics of CSAs Participating in the Research

	Location	Year est.	Governance model	# of members	# of participants	Median age	Median income*
**CSA 1**	SW Wales	2018	Family business	21	15	42	£35,714
**CSA 2**	SW Wales	2010/ 2018	Workers’ cooperative	66	6	40	£36,654
**CSA 3**	East Anglia	2012	Community Interest Company	65	12	49	£35,000
**CSA 4**	East Anglia	2008	Community Benefit Society	120	16	35	£29,851

* Participants' equivalized household disposable incomes, using the modified Organization for Economic Co-operation and Development (OECD) scale.
